# Time toxicity in cancer treatment: oncologists’ knowledge and practices across Pakistan

**DOI:** 10.3332/ecancer.2025.1957

**Published:** 2025-08-05

**Authors:** Muhammad Arif Hameed, Insia Ali, Waqas Ahmed Khan, Misbah Soomro, Mirza Rameez Samar, Yasmin Abdul Rashid, Tasneem Dawood

**Affiliations:** Medical Oncology, Department of Oncology, Aga Khan University Hospital, Stadium Road, Karachi 74600, Pakistan

**Keywords:** time toxicity, cancer treatment, cross-sectional study, medical oncologists, clinical hematologists, radiation oncologists, palliative care, tele-medicine

## Abstract

**Background:**

Time toxicity refers to the considerable time investment required by patients undergoing cancer treatment, including travel, waiting periods and treatment duration. It is increasingly recognised not only as a logistical burden but also as a psychological stressor, significantly affecting patient well-being.

**Objectives:**

This study surveyed oncologists across Pakistan to assess their understanding of time toxicity and its impact on both patients and clinical practice.

**Methods:**

From August to October 2023, we conducted a cross-sectional study targeting a diverse group of cancer care professionals including medical oncologists, clinical hematologists, radiation oncologists and palliative care physicians – across various healthcare centers in Pakistan. An online questionnaire was used to gather insights into their perspectives on time toxicity.

**Results:**

Over 54% of oncologists had a basic understanding of time toxicity, with 83.6% recognising its importance in cancer care. However, 69% noted that patients were poorly informed and often did not consider time burden in decision-making. About 45% of patients spent more than 10 hours per week on care-related activities. Key factors influencing time toxicity included cancer type, stage and logistical challenges. Notably, 85% of oncologists reported modifying treatment plans to reduce this burden. Strategies included offering chemotherapy services closer to patients’ homes, using telemedicine for consultations and proactively managing side effects. The psychological toll of time-consuming schedules was also acknowledged by respondents.

**Conclusion:**

Time toxicity poses a substantial burden in cancer treatment, both practically and psychologically. Enhancing healthcare providers’ awareness, improving access to care and adopting patient-centered approaches can significantly improve patient outcomes and quality of life.

## Introduction

While the treatment outcomes for various cancers have shown progress over time, for many incurable solid malignancies, the effectiveness of individual treatments is often limited and typically extends survival by an average of only a few months [1]. These benefits must be weighed against the potential negative effects of the treatments. A holistic approach to the care of cancer patients focuses on the complete well-being of patients, including social, psychological, emotional and spiritual aspects, alongside their physical health requirements [2]. Although oncologists often discuss potential side effects and financial aspects of care, they tend not to pay much attention to the time costs and usually do not incorporate the concept of ‘time toxicity’ and its impact into these conversations [3, 4].

Time toxicity refers to the adverse impact of the duration of cancer treatment, including procedures, therapies and the time spent away from home for travel and waiting, emergency visits, hospitalisations and managing other aspects such as finances and insurance [5]. This exhaustive time investment can significantly impact decision-making in cancer care, particularly in situations where the benefits of further treatment are small [6]. In response to the challenges of time toxicity, there is a growing emphasis on streamlining processes to lessen these time-related burdens.

Some of the prior studies have looked into the significant time investments for cancer care by patients and caregivers. In one study, patients diagnosed with metastatic pancreatic cancer undergoing systemic chemotherapy were found to spend, on average, about 10% of their time from diagnosis to death engaged in cancer treatment activities [7]. Meanwhile, those with lung cancer in their last year of life are estimated to utilise almost 600 hours (more than 24 days) in receiving care related to their cancer [8]. Additionally, caregivers for patients with advanced cancer are estimated to dedicate an average of 11 hours daily to providing care, a commitment that has been associated with a reduction in caregiver quality of life, effects of which can last for years following a family member’s cancer diagnosis [9].

In addition to the physical and logistical burdens, time toxicity has considerable psychological effects on patients and their families. Extended hours spent on hospital visits and infusions, as well as time away from day-to-day activities, contribute to anxiety, depression and a loss of autonomy [10]. As a result of the extensive demands associated with cancer care, caregivers themselves often suffer from emotional exhaustion and sustained psychological strain, with studies reporting elevated levels of caregiver burden and post-traumatic stress symptoms in some situations [11]. This time-related strain risks fracturing family dynamics, shortening the time spent with loved ones and fostering social isolation and financial suffering from missed workplace or caregiving demands [12]. The cumulative stress of balancing life with continuous medical obligations can erode patients’ overall quality of life and disrupt caregivers’ lives as well [13].

As the healthcare landscape shifts towards a model of shared decision-making, it is crucial to recognise the significant time investment required by patients and caregivers for treatments, especially when the outcomes are uncertain. Understanding the views of patients and their families as well as their care providers on time investment in cancer care is crucial for adopting efficient, patient-centric approaches, to improve patient well-being and quality of life [14]. Effective management of time toxicity can potentially enhance patient outcomes and adherence to treatment plans. By balancing treatment efficacy with patient convenience, we can improve patient satisfaction and overall outcomes in cancer care. The approach moves away from focusing solely on advanced treatments to enhancing the practical elements of everyday clinical care.

In this study, we sought to grasp physicians’ perception of the concept of time toxicity, their understanding of its significance and their views on the necessity of recognising and addressing it. We aimed to explore the methods they believe could prove effective in managing this problem, to acquire a more profound understanding of the appropriate actions to be taken.

## Methods

This study was conducted across multiple healthcare centers in Pakistan, targeting a wide range of oncology specialists and trainees, including those with expertise in medical oncology, clinical hematology, radiation oncology and palliative medicine. These centers varied in size and resources, offering a comprehensive overview of the cancer and palliative care landscape in Pakistan. The primary focus was to assess the understanding and management of time toxicity in cancer treatment among these professionals.

We developed a 28-question survey (Appendix for full questionnaire), focusing specifically on the perception of the concept of time toxicity in cancer treatment. The questionnaire captured demographic data, professional experience and perspectives on time toxicity. This survey was informed by a review of existing literature on time toxicity and its impact on cancer treatment outcomes and patient care. It included both Likert-scale and open-ended questions. The survey was designed to cover a wide range of perspectives and insights from experts.

The survey was developed and shared using the SurveyMonkey platform and sent out through emails and WhatsApp messages to reach as many professionals as possible across Pakistan. We invited people to participate using our professional contacts and email lists and sent out regular reminders to encourage more responses.

The survey was structured as an exploratory study, and as such, it did not formulate any formal hypotheses. The findings from the survey are presented in the results section in a descriptive format, including the use of tables and graphs. The study also documented and emphasised the varying perceptions expressed by respondents, noting these observations within the research.

## Results

A total of 67 responses were collected, with a gender distribution of 53% male and 47% female. 54 respondents (80%) completed the entire survey, while 8 participants (12%) completed more than 80% of the survey. The rest of the responses were less than 80% complete. The majority of respondents (63%) indicated their workplace in the Sindh region, followed by Punjab at 19.3%, Khyber Pakhtunkhwa at 15.8% and Balochistan at 3.5% (Table 1). In terms of specialisation, 69.35% of participants identified themselves as medical oncologists, while the remaining respondents included clinical hematologists, palliative care physicians and radiation oncologists. Despite 71% of participants holding consultant or faculty-level positions, a significant portion had less than 5 years of experience (56.5%) in their respective fields, with only 22.6% having over 10 years of experience in the respective field. More than half of the participants (56%) reported their affiliation with private university hospitals.

Regarding the concept of time toxicity, the prevailing belief among respondents was that it includes the time dedicated to various medical interventions and investigations such as blood work, radiology or pathology. They also highlighted contributing factors, including the time spent away from home for treatment, appointments, medical tests and the challenges of managing both side effects and financial aspects. An overwhelming majority (82%) acknowledged the relevance of time toxicity to cancer patients as well as oncologists and palliative care physicians.

54.5% of respondents reported having a basic understanding of time toxicity, while 83.6% recognised its clinical significance in cancer care. This difference suggests that although many respondents may not be thoroughly familiar with the formal concept, they still acknowledge its relevance in clinical practice. The survey captured this distinction through separate questions assessing conceptual familiarity and perceived impact. A similar percentage of respondents reported encountering patients who frequently have to travel long distances for consultations and infusion treatments (Table 2).

Concerning patient education, a notable 69% of respondents indicated that their patients are generally not well-informed about the concept of time toxicity and its implications throughout their care journey. Regarding patient care practices, 40% of oncologists incorporate considerations related to time expenses when selecting treatment options for patients, while 57% view it as an essential factor in enhancing patients’ quality of life.

**Figure 1.**
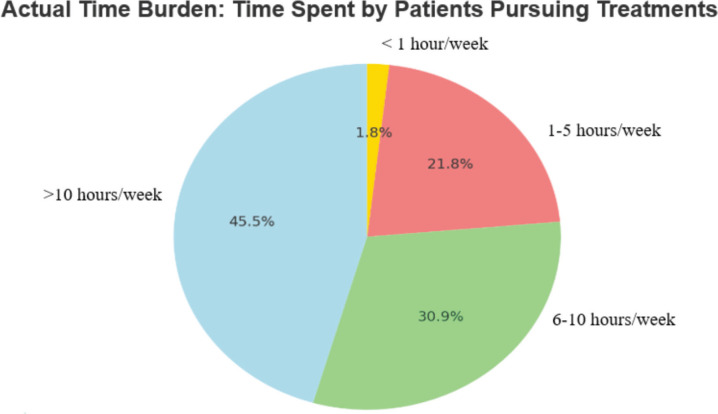
Actual time burden: time spent by patients pursuing treatments.

**Figure 2.**
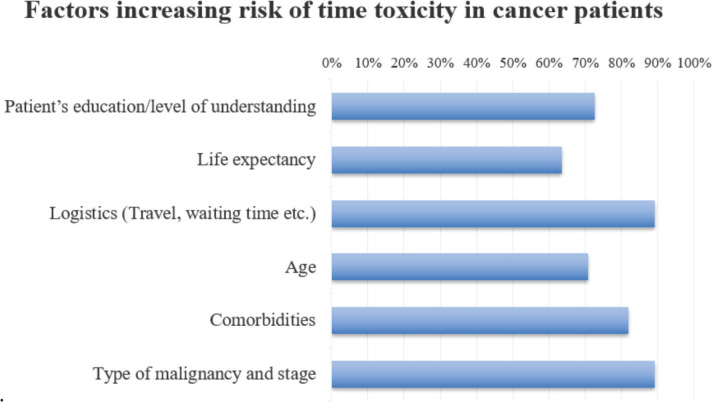
Factors contributing to time toxicity in cancer patients.

When asked about the actual time burden, 45% of respondents reported that their patients typically spend more than 10 hours per week on activities related to their cancer care, whereas 33% mentioned that the average time patients dedicate to cancer care falls within the range of 5–10 hours per week (Figure 1). When examining the factors contributing to time toxicity, respondents overwhelmingly identified the type and stage of malignancy as well as logistical challenges as the most significant contributors (Figure 2). Comorbidities and patient education about the disease followed closely, with concern levels at 82% and 73%, respectively. Impressively, 85% of respondents reported making modifications to patient treatment plans to accommodate the time constraints faced by patients, particularly those residing in remote areas. These adjustments included making infusions more convenient or less frequent (86%), reducing in-person clinic visits by implementing alternatives like tele-consultations (67%) and limiting the frequency of lab and radiology assessments (62%).

**Figure 3.**
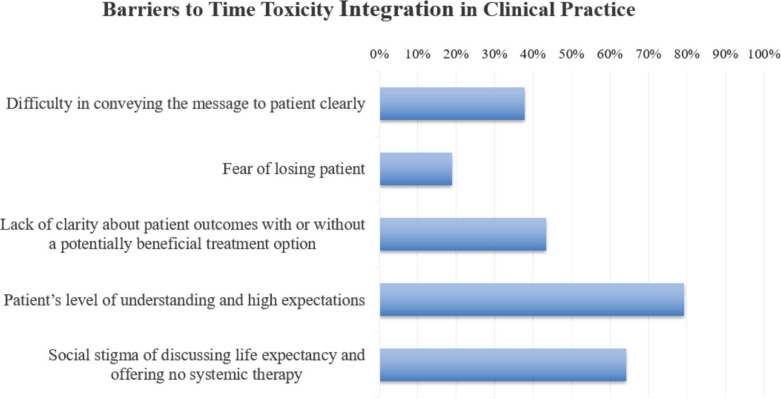
Barriers and challenges in integrating the concept of time toxicity to cancer care in Pakistan.

Furthermore, the study highlighted barriers to integrating discussions about time toxicity into clinical practice. 79% of participants identified the patient’s level of understanding and their high expectations as huge challenges, while 64.25% pointed to the social stigma associated with discussing life expectancy and the reluctance to withhold systemic therapy as major barriers (Figure 3). When it comes to incorporating these discussions into the consent process, 56% believed that in-person initial discussions were the most effective approach, while 32% favoured using written material.

**Figure 4.**
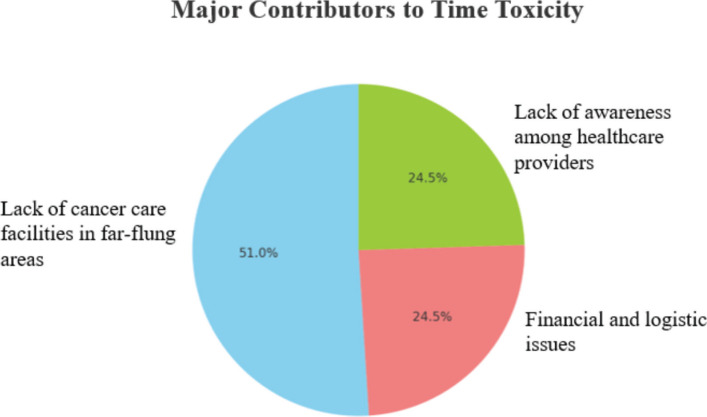
Major contributors to time toxicity.

**Figure 5.**
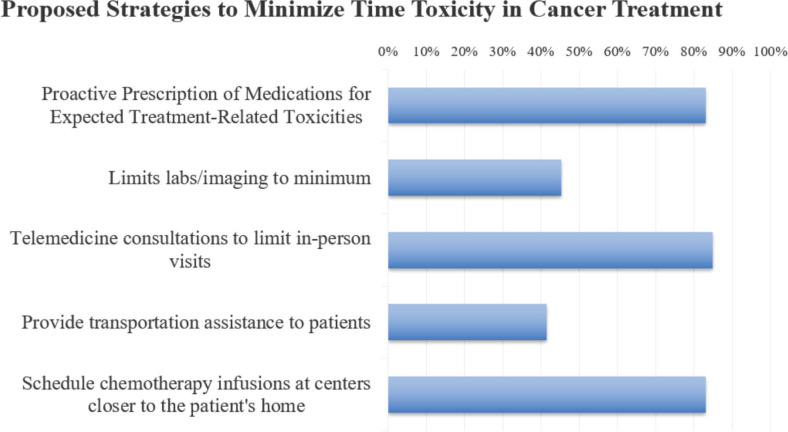
Oncologists’ evaluation of strategies to minimise time toxicity in cancer treatment.

Over half of the respondents considered the lack of cancer care facilities in remote areas as a major contributing factor, with other major factors being physicians’ lack of awareness regarding the consequences of time expenditures and logistical issues (Figure 4). Approximately 75% believed that improving physicians’ knowledge and awareness, along with enhancing access to cancer care facilities through the establishment of more cancer hospitals, improved transportation options and the promotion of telemedicine, would significantly enhance outcomes. Figure 5 shows that the top three strategies to reduce time toxicity in cancer treatment, as indicated by respondents, are scheduling chemotherapy infusions at centers closer to the patient’s home, utilising telemedicine consultations and adopting a proactive approach to managing expected adverse effects with detailed preemptive prescriptions. Additionally, 94% of respondents stressed the importance of conducting educational and awareness sessions on time toxicity for cancer care providers to enhance patient outcomes.

## Discussion

Cancer not only puts a physical and financial burden on patients but also demands considerable time for treatment, a factor that is frequently overlooked. This includes the time consumed in traveling, waiting for appointments and undergoing treatments and procedures, collectively referred to as ‘time toxicity.’ This burden not only affects patients’ routines but can also lead to psychological effects such as decision fatigue, helplessness or reduced treatment adherence, ultimately influencing their quality of life and decision-making [15]. Particularly for those with advanced cancer, the decision on how to allocate their remaining time and to balance the potential for extending their life with treatment against the desire to spend more time with their families becomes crucial. Recognising the importance of efficient time management in cancer care is vital for healthcare providers to ensure they offer patient-centered care that respects the value of time for patients and their loved ones. Previous studies reveal that discussions about time management are often overlooked, focusing instead on treatments, finances and outcomes. This approach leaves patients and their families unprepared for the rapid onset of end-of-life, emphasising the need to enhance communication about prognosis and life expectancy for better preparation and decision-making [16, 17].

In our study, a majority of physicians highlighted that time toxicity significantly burdens patients and their families, and this fact is often ignored in cancer care dialogues. Over 83% of clinicians frequently encounter patients who must spend extended periods away from home for treatments like chemotherapy and radiation therapy, indicating a significant impact on patients’ lives. While most clinicians acknowledge its impact, many noted limited patient education and variability in how the concept is applied in practice. This points to a need for improved communication and better integration of time considerations in cancer therapy. The observed gap between clinicians’ awareness of time toxicity and their clinical experiences suggests that many recognise its impact without necessarily using the formal term. We suggest directing educational approaches to refine the frameworks of time toxicity in clinical decision-making.

When evaluating the risk of extensive time costs in cancer patients, clinicians take into account a variety of factors. Critical considerations guiding treatment decisions include the type and stage of cancer, as well as the presence of patient comorbidities. Logistics, such as travel and waiting times, are also considered to minimise the burden on patients [18]. Additionally, a patient’s age, life expectancy and level of comprehension play significant roles in tailoring treatment plans to their individual circumstances. These considerations aim to strike a balance between effective treatment and the quality of life of an individual patient. Long travel times, especially for rural patients, may increase not only the objective time cost but also psychological fatigue and distress. These findings suggest a need for geographically sensitive interventions such as expanding telemedicine, decentralising services or setting up satellite clinics to reduce time and emotional burden.

In addition to financial and logistical challenges, factors like limited patient education, communication gaps and lack of formal training of physicians also add to the issue. Communication barriers play a major role, as illustrated in Figure 2. Several challenges hinder clear communication between doctors and cancer patients, as evident from survey responses: difficulty explaining treatment uncertainties and complexities, concerns about losing patients when discussing treatment limitations and discomfort with end-of-life topics. Oncologists in our survey emphasised the importance of integrating time toxicity education early in cancer care, particularly during the diagnosis and initial treatment planning phases. The proposed methods for educating patients about time toxicity included in-person consultations and the provision of written material to the patients [19]. A small minority of respondents suggested incorporating time toxicity education into the consent process, emphasising the need for patients to have a comprehensive grasp of treatment timelines. These findings highlight the need for enhanced patient education and communication to align expectations with realistic outcomes [20].

The majority of oncologists acknowledged the need for personalised strategies and to adapt patient-centered treatment plans to minimise time toxicity, recognising its growing importance in clinical practice. The approach may include modifying treatment regimens by transitioning patients to medications that are easier to administer or require less frequent dosing schedules. Over two-thirds of respondents believed tele-consultations could minimise time toxicity associated with in-person clinic visits, thereby reducing the need for them. This shift aligns with recent trends, especially post-COVID-19 pandemic, that favour virtual care models to improve efficiency and patient comfort. Furthermore, 63% of survey participants emphasised limiting laboratory work, focusing solely on essential tests, while 44% advocated for reducing the frequency of periodic disease assessments, such as imaging and physical exams, to allocate healthcare resources more efficiently. This shift toward patient-centered care aims to simplify treatment, improve adherence and reduce potential complications associated with complex dosing schedules.

Our study proposes holistic strategies to manage time toxicity in cancer care, aiming to enhance the entire care system. Key strategies include improving access to well-equipped cancer care facilities, particularly in remote areas, through new hospitals, clinics or enhanced transportation and financial support for patients. Telemedicine is also crucial, particularly for outpatient care, enabling patients to manage medication, toxicity and receive counseling remotely, thus reducing travel. Additionally, boosting physician awareness of time management in patient care through educational sessions and workshops is essential. These strategies, strongly supported by survey respondents, aim to significantly improve patient-centered care by addressing time toxicity efficiently.

To address time toxicity in cancer care effectively, particularly in regions like Pakistan, it is essential to enhance physicians’ awareness and understanding of this issue with a special focus on those dealing with cancer patients. Developing comprehensive educational programs for oncologists and healthcare providers is essential. These programs should focus on the concept of time toxicity, highlighting how treatment time impacts patients’ quality of life. Cultural beliefs and system barriers in Pakistan like reluctance to engage in end-of-life care discussions could also hinder open conversations about time burden. Incorporation of this topic into oncology curricula will sensitise future clinicians regarding its importance and could lead to more honest and effective conversations with patients.

Conducting local research to collect data on time toxicity will support evidence-based strategies and emphasise the significance of this issue. Establishing platforms for direct dialogue between oncologists and patients will facilitate the collection of patient-centered and real-world data, improving both patient care and our understanding of time toxicity. Participating in community outreach programs where oncologists educate the public and other clinicians about time toxicity and its impacts will help build a broader understanding and supportive network for patients.

Moreover, when designing and conducting clinical trials, it is important for oncologists and researchers to consider the impact of time toxicity on patient participation, adherence and outcomes. This approach will allow for a more thorough examination of how individual treatments affect patients’ time, providing fresh insights and enhancing understanding of the best practices in cancer care.

By focusing on these strategies, oncologists and related healthcare providers can gain heightened awareness of the challenges posed by time toxicity in cancer care, ultimately leading to more effective and empathetic treatment approaches. A stronger focus on reducing time-related burdens can lead to better outcomes and improved patient quality of life.

## Limitations

The exploratory and qualitative nature of this study, as well as the absence of formal hypothesis testing, naturally comes with limitations. However, the distribution may cause a response bias, as the majority of responses (63%) were obtained from Sindh alone, and hence, oncologists from other provinces and sub-specialties of oncology in Pakistan may be relatively under-represented. Furthermore, although a considerably large number of respondents reported seeing patients with a high burden of travel, there was no specific data collection regarding different geographic or socio-economic factors at the patient level (e.g., rural versus urban). This, consequently, hinders our understanding of how time toxicity across demographic groups.

In addition, self-reported data in the study could also lead to response bias as respondents may describe perceptions skewed by individual experiences and recall. The cross-sectional design further limits our capacity to explore changes or fluctuations in perceptions of time toxicity over time. Finally, non-responders may have very different views from responders, introducing a potential bias in response rates that could hinder the generalisability of the results.

Despite these limitations, the study presents some preliminary and useful insights into the perspectives of oncologists regarding time toxicity in cancer care in Pakistan. It is a stepping stone for more powerful research efforts in the future. Further studies that incorporate broader quantitative measures, longitudinal designs and clinical trials are encouraged to gain a deeper understanding and validate these initial findings. Such efforts would greatly inform patient education initiatives and guide efforts to reduce time toxicity in treatment planning, particularly in resource-limited settings.

## Conclusion

By improving access to cancer care through telemedicine, raising doctors’ awareness of time toxicity and focusing on patient-centered strategies, we can significantly reduce the impact of time toxicity on patients. Reducing treatment durations and enhancing communication about time-related burdens, prognosis and treatment options will help patients make informed decisions that reflect their values and reduce disruptions to their everyday lives.

## Conflicts of interest

There are no conflicts of interest.

## Funding

No specific funding has been used for manuscript writing or reporting.

## Ethical approval

This study was approved by the Institutional Ethics Review Committee (ERC No. 2023-8922-25524).

## Availability of data and materials

The data that support the findings of this study are available from the corresponding author upon reasonable request.

## Informed consent

Not applicable.

## Author contributions

MAH, IA, WAK and YAR drafted the initial manuscript. MAH organised and refined the content. MRS, MS and IA reviewed and gave feedback. YAR, TD and MAH approved the final version. TD, IA and MRS conducted data analysis. MAH handled the submission.

## Figures and Tables

**Table 1.** Demographics of study participants.

		Number of responses (*n*)	Percentage (%)
Gender	Male	33	53
	Female	29	47
Region	Sindh	36	63
	Punjab	11	19.3
	Baluchistan	2	3.5
	Khyber Pakhtunkhwa	9	15.8
Specialty	Medical Oncology	43	69.35
	Radiation Oncology	8	12.9
	Heme-Oncology	15	24.2
	Palliative Medicine	2	3.23
Current working position	Consultant/Faculty level	44	71
	Fellow	2	3.23
	Resident	15	24.2
Years of experience	Less than 5 years	35	56.45
	5–10 years	13	21
	More than 10 years	14	22.6
Types of practice	Private Practice	7	11.86
	University Hospital private setting	33	56
	University Hospital Government	15	25.4
	Tertiary care under an NGO	4	6.8

**Table 2.** Survey summary: oncologists’ perspectives on time toxicity in cancer care in Pakistan.

Question	Key findings	*N*	%
Physician’s understanding of the concept of time toxicity in cancer patients	Well versed with the concept	11	20
	Have an idea but not very well versed	30	54.5
	Very new to the concept	14	25.5
Understanding of clinical relevance of the concept	Yes	46	83.6
	No/ Not sure	9	16.4
Frequency of encountering patients receiving treatment away from usual place of residence	Often	46	83.6
	Occasionally/rarely	9	16.4
Patients’ level of awareness when they are receiving treatment	Somewhat aware/not considered	17	31
	Patient is not aware	38	69
Impact of time toxicity on quality of life of patients	Extremely important/Very important	49	91
	Somewhat important	5	9
	Not important	0	0
Actual time burden: time spent by patients pursuing treatments, including travel time and waiting time at cancer care facilities	More than 10 hours per week	25	45.5
	6–10 hours per week	17	30.9
	1–5 hours per week	12	21.8
	Less than 1 hour per week	1	1.82
Treatment modifications based on potential time toxicity	Yes	44	80
	No	11	20
Preferred strategies for modifying care plan	Alternate/convenient treatment plan	37	86
	Reduced lab/radiology work up	27	63
	Less frequent disease assessments	19	44
	Reduced physical follow ups	29	67
Proposed methods for effective communication to patients	Providing written materials	17	32
	Discussing during in-person consultations	30	56.6
	Including the concept in the chemotherapy consent form	6	10
Major contributors to time toxicity	Lack of cancer care facilities in far-flung areas	27	51
	Lack of financial resources to cover travel and accommodation costs	13	24.5
	Lack of awareness among healthcare providers about the concept of time toxicity	13	24.5
How to address cancer related time toxicity at large	By improving access to cancer care facilities	39	75
	By offering more support services to patients, such as transportation and accommodation assistance	34	65.4
	By incorporating telemedicine into cancer care delivery	30	57.7
	By increasing awareness among healthcare providers about the concept of time toxicity	38	73
Need for educational sessions on the topic	Yes	50	94.3

## Appendix: Survey questionnaire

**This survey questionnaire was sent to medical professionals through Email and Whatsapp messages via the following link:** https://www.surveymonkey.com/r/59GFPQF **.**

**Understanding of the concept of time toxicity in cancer treatment among oncologists in Pakistan**

*This survey assesses physicians’ knowledge and understanding of time toxicity in cancer therapy and explores the extent to which they consider this concept in their clinical practice. Your participation in this survey is completely voluntary and your responses will be kept confidential. The survey will take approximately 10–15 minutes to complete.*

**Age**: ______ years

**Gender**: Male □ Female □

**Region of Practice**: Sindh □ Punjab □ Baluchistan □ KPK □ Islamabad □

**Specialty:** Medical Oncology □ Radiation Oncology □ Heme-Oncology □

**Level of Practice: **Qualified Oncologist □ Fellow in Training □ Resident in Training □

**Experience **__________ years

**Type of Practice: **Private clinic □ Private Hospital □ Academic Hospital □ Government Hospital □

**1. When speaking of time toxicity what comes to your mind?**

*** Select as many as applicable***

□ Financial toxicity

□ Time spent in the hospital

□ Time spent getting second opinions for treatment

□ Deteriorating Quality of Life of Cancer patient

□ Time spent waiting for appointments

□ Time spent in investigations, such as blood work, radiology or pathology

**2. Which statement best suits time toxicity?**

□ Time spent by patients arranging finances for the cancer care

□ Time taken for diagnosis and treatment of cancer

□ Time spent in dealing with side effects of treatment, including inpatient admissions

□ Time spent by patients away from home, so that they can get treatment for cancer and time spent for doctors’ appointments, investigations and treatment, including management of side effects and arrangement for finances.

**3. Time Toxicity is related to**

□ Palliative care physicians

□ Oncologist

□ Cancer patients

□ All of the above

**4. How do you rate your knowledge of the concept of time toxicity in cancer therapy?**

□ Well versed with the concept

□ Have an idea but not very well versed

□ Barely heard of it

□ This is the first time I have read this phrase

**5. Do you think time toxicity is a clinically relevant concept in cancer therapy?**

□ Yes

□ No

□ I’m not sure

**6. How often do you encounter cancer patients who must stay away from their usual place of residence for several weeks to receive chemotherapy infusions or Radiation therapy?**

□ Very often

□ Occasionally

□ Rarely

□ Never encountered such patients

**7. Do you think that patients are adequately informed about the concept of time toxicity before starting treatment?**

□ Yes

□ No

□ Not sure

**8. How often do you consider the concept of time toxicity in your decision-making for cancer patients?**

□ Very often, almost consider in all patients

□ For carefully selected patients

□ Once in a while

□ Never

**9. In your opinion, how much time do cancer patients in Pakistan typically spend pursuing treatments, including travel time and waiting time at cancer care facilities?**

□ Less than 1 hour per week

□ 1-5 hours per week

□ 6-10 hours per week

□ More than 10 hours per week

**10. In your opinion, how does time toxicity impact the quality of life of cancer patients?**

□ Significantly

□ Moderately

□ Slightly

□ Not at all

**11. What do you think are the potential implications of time toxicity for cancer patients?**

□ Poor patient compliance

□ Poor clinical outcomes

□ Patient-physician mistrust

□ Poor Quality of life

□ Other

**12. What do you think is the most significant impact of time toxicity on cancer patients in Pakistan?**

□ Causes financial burden

□ Causes emotional burden

□ Causes physical burden

□ Other

**13. What factors do you consider when assessing the risk of time toxicity in cancer patients? Select as many options as applicable**

□ Type of malignancy and stage

□ Comorbidities

□ Age

□ Logistics (Travel, waiting time and so on)

□ Life expectancy

□ Patient’s education/level of understanding

□ Other

**14. Have you ever adjusted a patient’s treatment plan based on the risk of time toxicity?**

□Yes

□ No

* If yes, then the modification was based on what factors*

□ Substituted treatment regimen, for something more conveniently or less frequently administered drug

□ Reduced clinic visits (e.g., by arranging more frequent tele-Consultations)

□ Limited laboratory work up to the minimum required

□ Reduced frequency of disease assessment (e.g., imaging, physical examination and so on)

**15. What barriers do you face when trying to integrate the concept of time toxicity into your clinical practice?**

*** Select as many as applicable***

□ Social stigma of discussing life expectancy and offering no systemic therapy

□ Patient’s level of understanding and high expectations

□ Lack of clarity about patient outcomes with or without a potentially beneficial treatment option

□ Fear of losing patient

□ Difficulty in conveying the message to patient clearly

□ Others

**16. How can the concept of time toxicity be better integrated into the informed consent process for cancer patients?**

□ By providing written materials explaining the concept

□ By discussing the concept during in-person consultations

□ By including the concept in the chemotherapy consent form

□ Other

**17. What factors do you think contribute to the high prevalence of time toxicity in cancer patients in Pakistan?**

*** Select as many as applicable***

□ Lack of cancer care facilities in far-flung areas

□ Limited access to transportation

□ Lack of financial resources to cover travel and accommodation costs

□ Lack of awareness among healthcare providers about the concept of time toxicity

□ Other

**18. How do you think the problem of time toxicity can be better addressed in cancer care in Pakistan?**

□ By improving access to cancer care facilities

□ By offering more support services to patients, such as transportation and accommodation assistance

□ By incorporating telemedicine into cancer care delivery

□ By increasing awareness among healthcare providers about the concept of time toxicity

□ Other

**19. Which of the following strategies do you think will be most important in reducing time toxicity?**

□ Schedule chemotherapy infusions at centers closer to the patient’s home

□ Provide transportation assistance to patients

□ Offer telemedicine consultations to reduce the need for in-person visits

□ Limits labs/imaging as much as possible

□ Prescribe medications to manage treatment-related symptoms at home

□ Others

**20. Do you think there should be educational sessions for physicians in oncology and research on time toxicity in cancer patients in Pakistan?**

□ Yes

□ No

□ I don’t know

*Thank you for your participation in this survey. Your responses will help us better understand the concept of time toxicity in cancer care and identify strategies to improve patient outcomes in Pakistan.*
